# Supplementary material for: Content-rich biological network constructed by mining PubMed abstracts
Source: BMC Bioinformatics. 2004 Oct 8;5:147. doi: 10.1186/1471-2105-5-147 (PMC528731; doi:10.1186/1471-2105-5-147)
Supplement: Additional File 2 — The original results of the above study (non-essential files are deleted to keep the file size under the limit set by BMC bioinformatics). [file 1471-2105-5-147-S2.bz2 › chilibotAdditionalFile2/dip20/89ID7962001E326/html/RECO_RECR.html]

 


 **RECO** and **RECR** 
  
Found 42 abstracts in PubMed, retrieved 20.  
 

 What does Google say? 
 PDF only 
| .edu only 

---

**Interactive relationship** (e.g. stimulation, inhibition, etc)

**Neutral relationship**- We therefore assigned  **recO**  to epistatic group alpha and predict that the  **RecO**  protein acts at the same stage of recombination as the RecF, RecL and  **RecR**  proteins, in a RecFLOR complex.  Ref: 10323239 Mol Gen Genet, 1999

**Non-interactive relationship** (e.g. studied together, co-existance, homology, etc.)

- RecF protein competes with  **RecO**  protein for  **RecR**  protein association and its addition destabilizes RecAOR filaments.  Ref: 11743007 EMBO J, 2001
- We analyzed effects of overexpression of RecE and RecT on illegitimate recombination during prophage induction in Escherichia coli and found that frequencies of spontaneous and UV induced illegitimate recombination are enhanced by coexpression of RecE and RecT in the wild type, but the enhanced recombination was reduced by recJ,  **recO** , or  **recR**  mutation.  Ref: 12169595 J Bacteriol, 2002
- In Escherichia coli, the recovery of replication following UV induced DNA damage is dependent upon several proteins in the recF pathway, including RecF,  **RecO** , and  **RecR** .  Ref: 10589843 Mol Gen Genet, 1999
- Expression of a gene s from the nin region of phage lambda partially complemented both the viability and recombination defects of the recF,  **recO** , and  **recR**  mutants and the recombination defect of ruvC but not of ruvAB or recQ mutants.  Ref: 10735883 J Bacteriol, 2000
- ii this pathway s requires functional recA, recF,  **recO** , and  **recR**  genes, suggesting the mechanism to be daughter strand gap repair.  Ref: 11073901 J Bacteriol, 2000
- Gap repair genes recF,  **recO** , and  **recR**  were not required.  Ref: 10224240 Genetics, 1999
- The results indicated that RecET mediated illegitimate recombination depends on the functions of RecJ,  **RecO** , and  **RecR** , suggesting that the RecE and RecJ exonucleases play different roles in this recombination pathway.  Ref: 12169595 J Bacteriol, 2002
- The protective effect of plasmid pGam18 against radiation was completely abolished by mutations in the most RecF pathway genes recF, recJ,  **recR** ,  **recO** , recQ, recN, and ruvB.  Ref: 10368783 Genetika, 1999
- However, lexA Def  **recO**  or lexA Def  **recR**  strains, although unable to facilitate PriA pol II dependent restart, were able to perform pol V dependent readthrough.  Ref: 11929519 Mol Microbiol, 2002
- **RecR**  protein, which is known to stimulate  **RecO**  protein to facilitate SSB protein displacement by RecA protein, inhibits annealing by  **RecO**  protein, suggesting that  **RecR**  protein may regulate the choice between the DNA strand invasion versus annealing pathways.  Ref: 12438681 Proc Natl Acad Sci U S A, 2002
- Finally, examples are shown for in vitro reactions involving DNA, RecA,  **RecO** , RecF,  **RecR** , and SSB that lead to a further understanding of recombinational repair.  Ref: 11098470 Mol Biotechnol, 2000
- The  **recO**  mutation did not affect the sensitivity of recF,  **recR**  or recL cells to DNA damaging agents, increased the sensitivity of recU and addAB cells and abolished the DNA repair capacity of recH cells.  Ref: 10323239 Mol Gen Genet, 1999
- Expression of the Escherichia coli mutA mutator phenotype requires recA, recB, recC, ruvA, and ruvC gene, but not recD, recF,  **recO** , or  **recR**  genes.  Ref: 10671469 J Bacteriol, 2000
- This function is countered by the RecF  **RecO**  competition for association with the  **RecR**  protein.  Ref: 11743007 EMBO J, 2001
- Furthermore, we find that  **RecO**  and  **RecR**  significantly stimulate this recombination directed DNA replication reaction,.  Ref: 12667462 Mol Cell, 2003
- Resistance of E. coli strains to cisplatin as determined by plating experiments decreased in the following order parent PQ30 strain, polB >  **recO** ,  **recR** , recF > recB.  Ref: 12531023 DNA Repair Amst , 2002
- The Escherichia coli RecF,  **RecO**  and  **RecR**  pro teins have previously been implicated in bacterial recombinational DNA repair at DNA gaps.  Ref: 11743007 EMBO J, 2001
- The defects in restart attributed to mutations in recF,  **recO**  or  **recR**  were suppressed in a recA730 lexA Def strain expressing constitutively activated RecA RecA\*.  Ref: 11929519 Mol Microbiol, 2002
- ii In a recBC sbcA background, in which RecE mediated recombination is active, recA, recJ, recQ, recE, recT, recF,  **recO** , and  **recR**  mutations led to their accumulation.  Ref: 12718760 BMC Mol Biol< MedlineTA>BMC Mol Biol, 2002
- Genetic evidence suggests that the RecF,  **RecO** , and  **RecR**  RecFOR proteins participate in a common step of DNA recombination and repair, yet the biochemical event requiring collaboration of all three proteins is unknown.  Ref: 12769856 Mol Cell, 2003
- We compared the recombination proficiencies of recB1080,  **recO** ,  **recR** , and recF single mutants and recB1080  **recO** , recB1080  **recR** , and recB1080 recF double mutants.  Ref: 12618388 Genetics, 2003
- Similarly, recF,  **recO**  or  **recR**  mutations suppress the lethality of the Escherichia coli rep uvrD double mutant.  Ref: 12065426 EMBO J, 2002
- Although the MRF alpha 2 components are apparently not encoded by currently known homologous recombination genes such as recA, recF,  **recO** , and  **recR** , they may fulfill an important function in assembling replisomes on arrested replication forks and products of homologous strand exchange.  Ref: 11459960 Proc Natl Acad Sci U S A, 2001
- dnaC suppressors of priA overcome this inviability, especially when RecF,  **RecO**  or  **RecR**  is inactivated, indicating that RdgC avoids or counters a toxic effect of these proteins.  Ref: 12554673 EMBO J, 2003
- the  **RecO**  and  **RecR**  proteins also play important roles in the recombination.  Ref: 12169595 J Bacteriol, 2002
- To elucidate the role of several key proteins involved in this process, we have analysed the time dependence of replication restart in strains carrying a combination of mutations in lexA, recA, polB pol II, umuDC pol V, priA, dnaC, recF,  **recO**  or  **recR** .  Ref: 11929519 Mol Microbiol, 2002
- In a wild type, lexA background, mutations in recF,  **recO**  or  **recR**  block both pathways.  Ref: 11929519 Mol Microbiol, 2002
- Roles of RecJ,  **RecO** , and  **RecR**  in RecET mediated illegitimate recombination in Escherichia coli.  Ref: 12169595 J Bacteriol, 2002
- previously isolated mutations in B.subtilis recF, recL,  **recO**  and  **recR** , which belong to the same complementation group, all suppress the lethality of a pcrA mutation.  Ref: 12065426 EMBO J, 2002
- An enhanced function of the  **RecO**  and  **RecR**  proteins can thus be seen in vitro at the 5 ends of linear ssDNA that is not as evident in DNA gaps.  Ref: 11743007 EMBO J, 2001
- In this genetic background, mutations in recF,  **recO** , and  **recR**  had large effects on both cell viability and on recombination.  Ref: 10735883 J Bacteriol, 2000
